# MiR-199a family members are differentially regulated during physiological and pathological cardiac hypertrophy

**DOI:** 10.3389/fphys.2026.1833828

**Published:** 2026-08-11

**Authors:** Virginie Joris, Dorothée Marchand, Thomas Metzinger, Laurent Dumas, Hrag Esfahani, Olivier Feron, Sandrine Horman, Chantal Dessy

**Affiliations:** 1 Pole of Pharmacology and Therapeutics (FATH), Experimental and Clinical Research Institute (IREC), Université Catholique de Louvain, Brussels, Belgium; 2 Pole of Cardiovascular Research (CARD), Experimental and Clinical Research Institute (IREC), Université Catholique de Louvain, Brussels, Belgium

**Keywords:** cardiac adaptation, cardiomyocyte, endothelium, exercise, microRNA

## Abstract

Among potential molecular mechanisms supporting cardiovascular homeostasis, miRNAs (miRs) represent interesting candidates. We recently highlighted that miR-199a controls the Nitric oxide synthase/nitric oxide (NOS/NO) pathway in the endothelium and showed that both mature strands of miR-199a are overexpressed in heart and vessels from a mouse model of hypertension. Here we investigated the fate of miR-199a in heart and vessels from mice developing physiological or pathological cardiac hypertrophy. Our working hypothesis is that alterations in cell phenotypes driven by changes in miR-199a abundance account for cardiac and/or vascular adaptation to increased workload in patho-physiological contexts. C57BL/6J mice were given access to voluntary dynamic wheel training during 22 weeks or underwent transverse aortic constriction (TAC) surgery. As a result, those mice respectively developed physiological and pathological cardiac hypertrophy. An opposite modulation of miR-199a-3p and miR-199a-5p expression was observed in vessels and heart from running *vs* TAC mice which correlated with opposite regulation of Retinobastoma1 (RB1) in cardiac tissue. We show that miR-199a-3p upregulation, by indirectly modulating CCAAT/enhancer binding protein (C/EBP)β, promotes cardiomyocyte hypertrophy in the pathological settings, while its repression by exercise drives metabolic adaptations associated with physiological remodeling. Interestingly, miR-199a-5p repression also directly target Sirtuin 1 (Sirt-1) and Peroxisome proliferator-activated receptor coactivator (PGC1)α Taken together, our findings indicate that both mature arms of miR-199a act in tandem to drive morphologic and metabolic adaptations encountered in physiological and pathological cardiac hypertrophy.

## Highlights

MiR-199a-3p and -5p have been proposed as potential regulators of cardiovascular functions. Our present work shows that:

- miR-199a-3p and miR-199a-5p are inversely regulated in cardio-vascular tissue following pressure overload or exercise training
- miR-199a-3p is a key regulator of cardiomyocyte size, cardiac hypertrophy and metabolic adaptations through C/EBPβ in response to pressure overload or chronic exercise. The opposite strand, miR-199a-5p directly regulates PGC1α and Sirt-1 to promote mitochondrial biogenesis and oxidative metabolism in the context of exercise
- miR-199a is at the crossroads of physiological and pathological cardiac remodeling, driving a coordinated regulatory network involving multiple downstream effectors

## Introduction

The cardiovascular system continuously adjusts to ensure optimal organ perfusion under all conditions. In response to increased workload, these adaptations involve both functional and structural changes of the left ventricle leading to heart enlargement and development of hypertrophy. Depending on the initial stimuli, two distinct phenotypes can be observed, and categorized as physiological *vs* pathological. Physiological cardiac hypertrophy is the adaptative response often observed during pregnancy or in athletes. Chronic volume overload associated with dynamic endurance training induces eccentric cardiac remodeling characterized by its reversibility, symmetrical chamber dilation with proportional wall growth, and preserved or even enhanced cardiac function. At the vascular level, endothelial function is improved, while Vascular endothelial growth factor (VEGF) expression and angiogenic processes are upregulated to promote vascular expansion and maintain adequate perfusion to parallel myocardial growth. Pathological cardiac hypertrophy develops in response to pressure overload under chronic stress conditions such as hypertension. In this context, remodeling is concentric and becomes maladaptive, it is largely irreversible, characterized by disorganized myocardial growth and frequently impaired cardiac function. Endothelial function and angiogenic capacity are diminished or insufficient, leading to reduced perfusion efficiency and compromised tissue oxygenation. Numerous molecular and cellular events leading to physiological versus pathological cardiac adaptations have been described. They include the so-called “fetal gene” reprogramming that supports the switch in fuel metabolism and bioenergetics characteristic of maladaptive cardiac hypertrophy or the regulation of growth factors and signaling molecules involved cellular growth (Maillet et al., 2013; Nakamura and Sadoshima, 2018).

Among the possible molecular mechanisms that contribute to cardiovascular (CV) homeostasis is the modulation of microRNA (miRNA, miR) expression profile. These small non-coding RNAs regulate virtually all biological processes involved in homeostasis via a post-transcriptional control of gene expression (Van Rooij et al., 2006; Kukreja et al., 2011). Dysregulation of specific miRNAs profile has been associated with many pathophysiological states, and several pieces of evidence suggest that alterations in miR-199 expression could also tip the balance toward either CV disease (CVD) or exercise-mediated vasculo-protective effects. We and others have indeed identified miR-199a mature products as negative regulators of CV function (Song et al., 2010; Latronico and Condorelli, 2011; Heuslein et al., 2018; Joris et al., 2018). Although originally identified as an actor of carcinogenesis, miR-199a has been suggested as a master regulator of cardiac metabolism through its action on Sirtuin (SIRT)-1 and Peroxisome proliferator-activated receptor coactivator (PGC)-1α (Yan et al., 2021). A dysregulation of miR-199a has also been proposed in several CV pathologies, both in humans and animal models (Latronico and Condorelli, 2011). For instance, miR-199a up-regulation has been associated with hypertension and/or pathological myocardial hypertrophy evoked by trans-aortic abdominal constriction (Song et al., 2010) or angiotensin infusion (Joris et al., 2018). We have documented how conversely, a reduction in miR-199a is associated with an increase in Endothelial nitric oxide synthase (eNOS) activity/NO availability, thereby pointing to miR-199a as a key regulator of endothelial function (Joris et al., 2018). This specific finding was further supported by the work of Heuslein et al. reporting up-regulated miR-199a in the blood of peripheral arterial disease patients with intermittent claudication (Heuslein et al., 2018). Furthermore, the use of miR-199-sponge transgenic murine model to generate a cardiomyocyte-specific disruption of miR-199a, was shown to promote the development of physiological cardiac hypertrophy (Li et al., 2016).

In this study, we used murine models of dynamic training or pressure overload to test the hypothesis that vascular and cardiac phenotype adaptations are driven by changes in miR-199a abundance.

## Materials and methods

### Animal housing and exercise protocol

Four-weeks-old C57BL/6J mice (25 animals) were purchased from Janvier Labs (Le Genest-Saint-Isle, France) and housed 1 per cage in our local animal facility with a 12/12-hour night and day cycle with free access to both food and water. Their behavior and wellbeing were checked every day. At five weeks of age, animals were randomly assigned to a cage (Tecniplast, Décines-Charpieu, France) equipped or not with an activity wheel (Kit mini wheel and activity wheel with revolution counter, Intellibio, Nancy, France). Of note, endurance training began before full adulthood to prevent age-related remodeling during the 6–7 months protocol. Animals’ body weight was measured every two weeks, while run distance covered was recorded every two days for 22 weeks. This timeframe was selected to ensure that cardiac adaptations were stable and maintained over time (Pei et al., 2021; Jyothidasan et al., 2024). At the day of sacrifice, terminal anesthesia was performed by intraperitoneal injection of ketamine (100 mg/ml) and xylazine (20 mg/ml), blood heart and vessels were collected and frozen in liquid nitrogen for (mi)RNA profiling and Western Blotting analyses. It is noteworthy that since miR-199a-3p appears to take part in the estrogen regulatory network and male and female react differently to running exercise for instance, this study was performed on male mice only (Fu et al., 2018; Manzanares et al., 2019).

All experimental procedures and protocols were approved by the local Ethics Committee (Comité d’Ethique pour l’Expérimentation animale; 2016/UCL/MD/017), Secteur des Sciences de la Santé, Université Catholique de Louvain, according to National Care Regulation and Directive 2010/63/EU of European Parliament and of the Council.

### Animal housing and TAC protocol

Twelve to sixteen-week-old C57BL/6J male mice underwent transverse aortic constriction (TAC) to induce cardiac hypertrophy. Fully mature mice were chosen for this protocol to ensure reproducible hypertrophic response. Twenty minutes prior to surgery, the animals were treated with 0.1 mg/kg of buprenorphine. Then, mice were anaesthetized with an intraperitoneal injection of a mixture of ketamine (150 mg/kg) and xylazine (10 mg/kg) and were placed in a supine position. A surgical incision of 1mm was conducted on the right side of the sternum under a microscope to avoid injury to the mammary artery. A constrictive band was placed around the aorta, which was then tightened around 27-gauge blunted needle, subsequently removed. In the sham-operated mice, the ligature was not tightened. The chest was closed with a polypropylene 6.0 suture. Following surgery, the animals were treated with 0.1 mg/kg of buprenorphine. Doppler measurements of trans-stenotic gradients were performed at 2 days and 3 weeks post-surgery. Only TAC mice with a velocity higher than 2.8 m/s were included in the experiment.

### Tissue preparation

After 22 weeks of voluntary wheel running or 9 weeks of TAC surgery, whole heart and vessels were excised. All samples were stored at 4 °C for physiological measurement or at -80 °C until further processing for proteomics or RNA measurements. Heart and vessels cleaned from all fat and surrounding tissue were either snap frozen or directly used according to the desired experiment.

### Histological staining

Samples of LV were fixed in 4% neutral formaldehyde diluted in phosphate-buffered saline and were paraffin embedded. LV samples were then cut into 5µm sections, deparaffined and processed for routine hematoxylin/eosin staining and Sirius red staining. Briefly, the slides were incubated with a 0.1% Sirius Red solution dissolved in aqueous saturated picric acid for 1 hour, washed in acidified water (0.5% hydrogen chloride), dehydrated and mounted with DPX Mounting (ThermoFisher, Waltham, MS, USA, LAMB/DPX). Collagen content was red-stained.

### MiRNA extraction

MiRNA extraction from tissues was performed as previously described (Joris et al., 2018). Briefly, hearts and aortas of mice were suspended in homogenization buffer, supplemented with thioglycerol as recommended by the manufacturer. Lysis buffer and proteinase K were added to the samples and, after incubation for 10min at room temperature, loaded in the cartridges provided with the kit (Promega, Madison, WI, USA, AS1460).

### MiRNA reverse transcription and quantitative PCR

Reverse transcription and quantitative polymerase chain reaction (PCR) for miRNAs were performed following the miRCURY^®^ LNA^®^ miRNA PCR Handbook for individual PCR Assay provided by Qiagen. Primers used were the following: miR-199a-3p (YP00204536), miR-199a-5p (YP00204494), U6 (YP00203907), and miR-let-7i-5p (YP00204394). Neither the U6 nor the miR-let-7i is modulated due to exercise, the latter was used as housekeeping gene for vessels. All reagents were purchased from Qiagen (Hilden, Germany), and the procedures were performed in accordance with the manufacturer’s recommendations.

### MiRNA precursor reverse transcription and quantitative PCR

Reverse transcription and quantitative polymerase chain reaction (PCR) for pre-miRNAs were performed following the miRScript PCR System Handbook for precursor detection provided by Qiagen (Hilden, Germany). Primers used for pre-miR were the following: mir-199a-1 (MP00001365), mir-199a-2 (MP00001372), and miR-let-7i-5p (YP00204394). The latter was used as housekeeping gene for vessels, following the manufacturer’s recommendations and was not modified in each condition. All reagents were from Qiagen (Hilden, Germany).

### Neonatal cardiomyocytes isolation and transfection

Cardiomyocytes were isolated from 1 to 3 days old neonatal rats. Rats were sacrificed and hearts were harvested in cold HBSS (No Calcium, No Magnesium). Auricles are discarded and ventricles are opened, quickly rinsed in ethanol and placed in cold HBSS. Ventricles were cut in small pieces and incubated in Trypsin-HBSS solution (15mg/25ml) overnight at 4 °C under agitation. Hearts are then incubated during 5min at 37 °C in DMEM Glutamax (4,5g glucose) 10% FBS, 1% streptomycin/penicillin and 1% amphotericin B. Medium was removed, and heart pieces were incubated in collagenase type II (0,1% w/v in HBSS) at 37 °C under agitation during 5min. Supernatant was collected in a falcon on ice. Cycle of collagenase were repeated till disappearance of heart pieces. Solution containing cells was filtered on cell strainer (40µm, VWR, Radnor, PA, USA, 732-2757) and centrifuged during 10min at 150g. Cells were incubated for pre-plating during 2h in a T175 flask to remove fibroblasts. Supernatant was collected and cells were counted using trypan blue. Cells were plated in 12-wells or 6- wells plates (250000 cells/cm2) in DMEM FBS 10%, pen/strep 1%, amphotericin B 1% and ARA C 10µM ON at 37 °C 5% CO_2_. After 24h, ARA C was removed. Transfection with 50nM siRNA ON-TARGETplus Rat Rb1 (Horizon Discovery, Waterbeach, UK, L-087650-02-0005), Silencer™ Pre-Designed siRNA mTOR (ThermoFisher, Waltham, MS, USA, AM16708) or ON-TARGETplus Non-targeting Control Pool (Horizon Discovery, Waterbeach, UK, D-001810-10-05); or miR-199a-3p (Qiagen, Hilden, Germany, YM00472279-ADA) or miR-199a-5p (Qiagen, Hilden, Germany, YM00471589-BDI) mimic or negative control (Qiagen, Hilden, Germany, YM00479902-BDI); or inhibitor of miR-199a-3p (Qiagen, Hilden, Germany, YI04100433-ADA) or miR-199a-5p (Qiagen, Hilden, Germany, YI04101096-ADA) or negative control (Qiagen, Hilden, Germany, YI00199006-ADA) was performed at this moment using Lipofectamine RNAiMAX (ThermoFisher, Waltham, MS, USA, 13778075). Cells were then cultured in DMEM/FBS10% for still one day. Medium is changed every day using DMEM supplemented with 2% Horse Serum and 0,05% HEPES to induce differentiation for at least 5 days. Beating of cardiac cells reflects a good quality of isolation. Cells were harvested for protein or miR analysis 5 days after transfection.

### Pull down assay

Pull down assay was performed on neonatal cardiomyocytes. Cells were cultured and transfected with a biotinylated mimic of miR-199a-5p as described above. Following a 24-hour incubation period, the cells were harvested in lysis buffer (20 mM Tris-Base; pH 7.5, 100 mM KCl and 5 mM MgCl_2_) supplemented with 0.05% Tween 20, 50 U RNAse OUT (ThermoFisher, Waltham, MS, USA, 10777019) and a proteinase inhibitor cocktail (PIC, ThermoFisher, Waltham, MS, USA, 78438). Prior to the pull-down, 150 µl of Dynabeads M-270 streptavidin (Invitrogen, Waltham, MA, USA, 65305) were washed three times with lysis buffer. The beads were blocked for a period of two hours under agitation at 4 °C in 800 µl of lysis buffer, which was supplemented with 0.05% Tween 20, 50 U RNAse OUT, PIC, 200 yeast tRNA (1 mg/ml, Invitrogen, Waltham, MA, USA, AM7119), and BSA 1%. Following two washes, 150 µl of harvested cells were incubated with the beads for four hours at 4 °C under agitation. The beads were then washed three times in 500 µl of lysis buffer and resuspended in TRIzol LS (Invitrogen, Waltham, MA, USA, 10296010) and extracted for RNA following the manufacturer’s protocol.

### RNA reverse transcription and quantitative PCR

Isolation of total RNAs was performed as described for microRNAs using the same method with Maxwell technology (Promega, Madison, WI, USA, AS1460). All reagent for RT-qPCR were purchased from ThermoFisher (Waltham, MS, USA), following the manufacturer’s protocol.

### Western blot

Tissues were suspended in RIPA lysis buffer. Protein concentration of each sample was determined by using a Bicinchoninic Acid Protein Assay Kit (ThermoFisher, Waltham, MS, USA, 23228) according to the manufacturer’s protocol. Western blot was realized as previously described (Joris et al., 2018). The primary antibodies that were used were the following: anti-phosphoSer1177eNOS (1µl/ml; Millipore, 07-428), anti-phosphoThr308Akt (1µl/ml; Cell Signaling Technology, Danvers, MA, USA, 2965), anti-eNOS (0,25µg/ml, BD transduction, 610297), anti-Akt (1µl/ml; Cell Signaling Technology, Danvers, MA, USA, 9272), anti-Twist1 (2µl/ml, Abcam, Cambridge, UK, ab50581), anti-Sirtuin1 (1µl/ml, Cell Signaling Technology, Danvers, MA, USA, 9475), anti-PGC1α (1µ/ml, Abcam, Cambridge, UK, ab54481), anti-mTOR (1µl/ml, Cell Signaling Technology, Danvers, MA, USA, 2983) anti-phosphoSer473 Akt (1µl/ml, Cell Signaling Technology, Danvers, MA, USA, 4060), anti-Rb (1µ/ml, Abcam, Cambridge, UK, ab181616), anti-C/EBPβ (1µ/ml, Abcam, Cambridge, UK, ab32358), anti-GAPDH (0,1µl/ml; Cell Signaling Technology, Danvers, MA, USA, 2118) and anti-βactin (0.02µl/ml, Sigma-Aldrich, Saint-Louis, MO, USA, A5441). Levels of all proteins of interest were normalized to the housekeeping proteins β-actin for vessels or GAPDH for heart tissue. The levels of phosphorylated proteins were normalized to the level of total proteins.

### Immunofluorescence

Cardiomyocytes were plated on laminin-coated slides at a density of 50–000 cells/1mL and cultured as described before. Cells were fixed in PFA 4% and later incubated overnight with α-actinin primary antibody (Sigma-Aldrich, Saint-Louis, MO, USA, A7811), followed the next day with the secondary antibody goat anti-mouse (Invitrogen, Waltham, MA, USA, A11031) and DAPI (Invitrogen, Waltham, MA, USA, D1306) incubation. Finally, and after a last washing with PBS, slides were mounted on microscope slides using Dako fluorescent mounting medium (Agilent, Santa Clara, CA, USA, S3023). Cardiomyocyte morphology was visualized using α-actinin immunostaining, and images were acquired by confocal microscopy using identical acquisition settings for all samples. Pictures were taken using microscope Zeiss AxioImager and cells were selected for analysis based on clearly defined sarcomeric α-actinin patterns, intact morphology, and the absence of overlapping or truncated structures. A minimum of 30 cardiomyocytes per slide were quantified using Fiji. Image analysis was performed without blinding.

### Statistics

All experiments were reproduced at least three times. All data are expressed as the Mean ± SEM. Shapiro-Wilk normality test was performed, and statistically significant differences were determined using a 2-way ANOVA with subsequent Bonferroni’s *post-hoc* analysis test. For two groups’ comparison, t-test student was used for parametric data or Mann-Whitney test for nonparametric data. P<0.05 was considered statistically significant.

## Results

### Pressure overload and dynamic training promote pathological and physiological cardiac hypertrophy respectively

**Figure 1:**
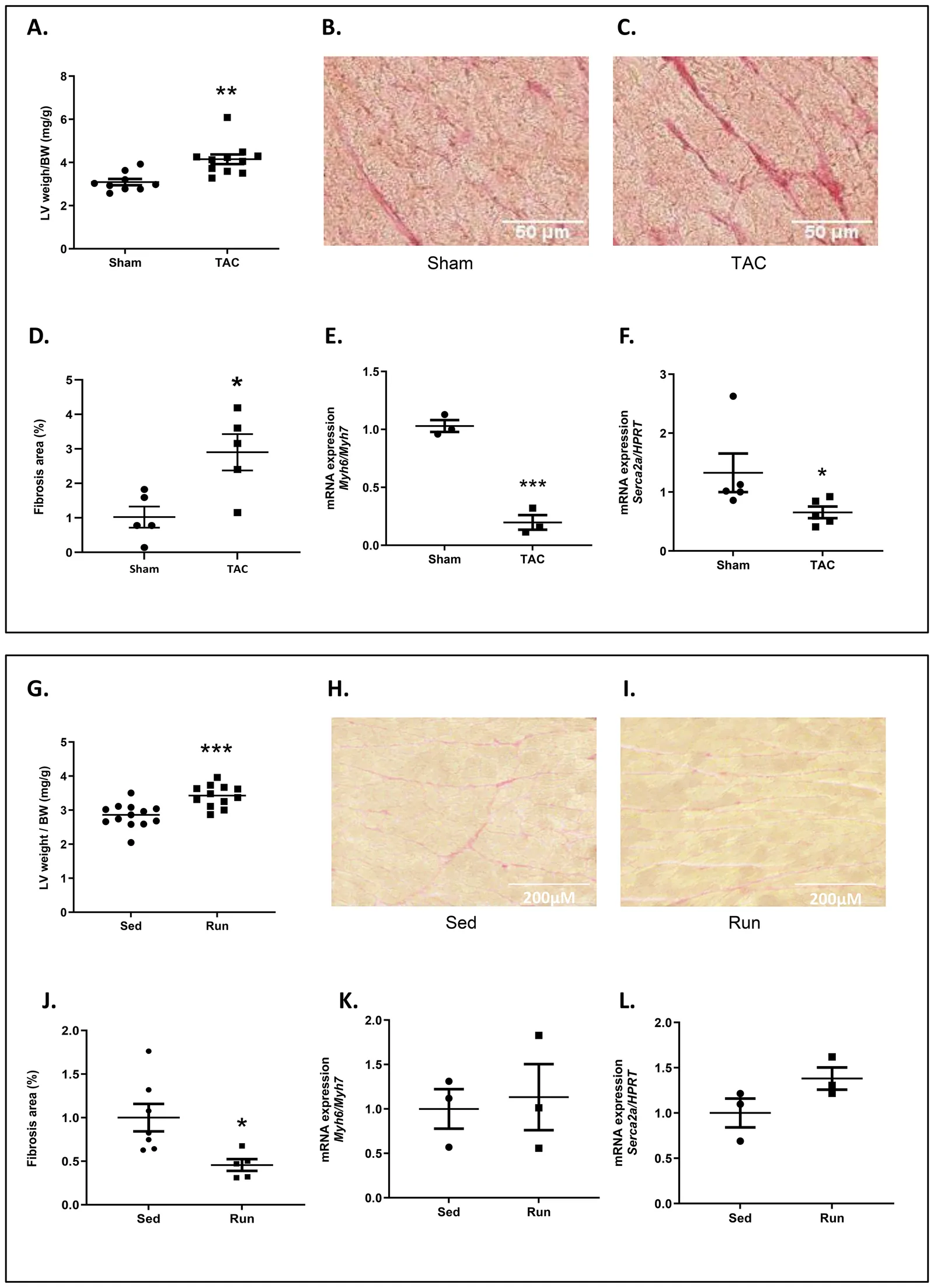
Hearts isolated from TAC mice (upper panel) and running mice (lower panel) respectively exhibit pathological and physiological hypertrophy. Left ventricular weight was measured and normalized on body weight for both TAC **(A)** and running mice **(G)**. Fibrosis area was measured for both TAC **(D)** and running **(J)** mice following a Sirius Red staining **(B, C, H, I)**. Myh6, Myh7 **(E, K)** and Serca2a **(F, L)** mRNAs levels were measured using RT-qPCR and normalized by HPRT expression. Results are expressed as mean ± SEM of minimum 3 animals. *P. < 0.05, **P. < 0.01 and ***P. < 0,001 vs. sham mice or sedentary mice (t-test or Mann-Whitney when appropriated). LV, left ventricle; BW, body weight; TAC, transaortic constriction; sed, sedentary; run, running mice.

After 8 weeks, mice subjected to transverse aortic constriction (TAC) developed marked cardiac hypertrophy, as evidenced by an increased left ventricle (LV) weight-to-body weight ratio (**Figure 1A**). This hypertrophic remodeling was accompanied by a higher myocardial collagen content (**Figures 1B–D**) and by molecular changes consistent with fetal gene reprogramming, including a decreased ratio of adult (*Myh6*) to fetal (*Myh7*) myosin heavy chain mRNA expression and a downregulation of the calcium-handling gene *Serca2a* (**Figures 1E, F**). In parallel, *ANP* mRNA expression was significantly increased, while *BNP* expression remained unchanged (**Supplementary Figures 1A, B**).

In parallel, a murine model of physiological cardiac hypertrophy was established. Five-week-old C57BL/6J male mice were housed in cages with free access to a running wheel for 22 weeks. Adaptation of the cardiac muscle to chronic exercise was evaluated by echocardiography along the training period. As body mass differed between active and sedentary mice, cardiac morphometric parameters were normalized on body weight. (see **Supplementary Table 1**). The average daily running distance increased during the first weeks, peaking at week 4 (7.17 ± 0.66 km), and progressively declined until week 10, as previously described (De Bono et al., 2006). From this timepoint onward, the mean distance remained stable at approximately 3.30 ± 0.21 km per 24 hours (**Supplementary Figure 1C**). From week 10 until the end of the protocol (week 22), running/exercise trained mice gained significantly less body weight than sedentary controls (**Supplementary Figure 1D**). After 22 weeks, exercise-trained mice exhibited significant cardiac hypertrophy compared with sedentary mice, as demonstrated by a significant increase in the heart weight-to-body weight ratio (**Figure 1G**) with left ventricular wall thickening in systole and diastole (**Supplementary Table 1**). Sirius Red staining of LV sections revealed mild but significant deposits of collagen in the heart of sedentary mice, which were barely noticeable in exercised animals (**Figures 1H–J**). At the molecular level, voluntary dynamic training induced a robust upregulation of cardiac *ANP* and *BNP* mRNA expression (**Supplementary Figures 1E, F**), while neither the *Myh6/Myh7* ratio nor *Serca2a* expression were significantly affected (**Figures 1K, L**) confirming that moderate chronic exercise was associated with a physiological cardiac hypertrophy.

### miR-199a-3p and miR-199a-5p are inversely regulated in vascular and cardiac tissue following pressure overload or exercise training

**Figure 2:**
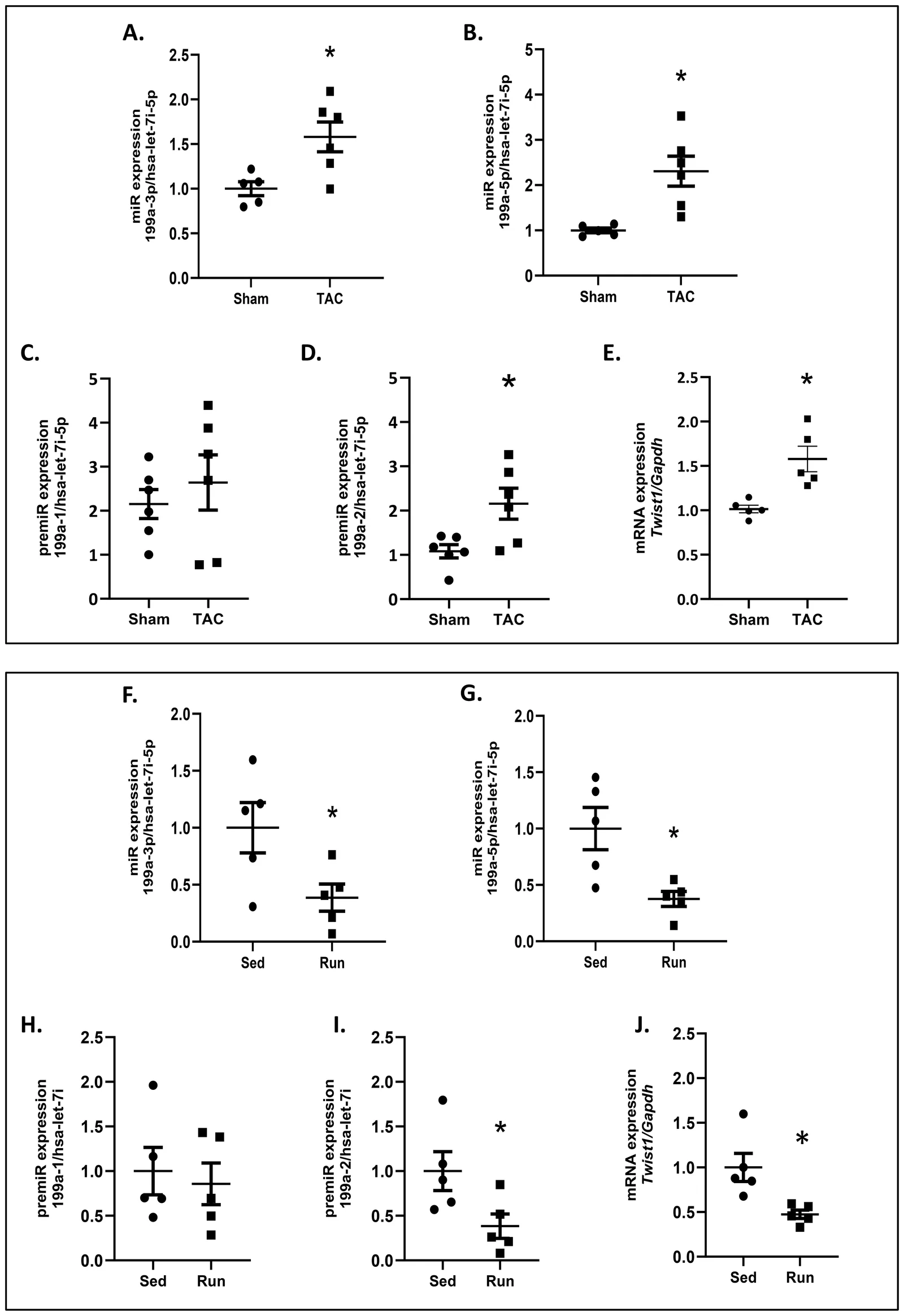
Mesenteric arteries isolated from TAC mice (upper panel) and running mice (lower panel) exhibit opposite microRNAs 199a expression. MiR-199a-3p and miR-199a-5p expression were measured in vessels from TAC **(A, B)** and running mice **(F, G)** using RT-qPCR. PremiRs and Twist1 expression were measured in vessels from TAC **(C–E)** and running mice **(H–J)** using RT-qPCR. Results are expressed as mean ± SEM of 5–6 animals. *P. < 0.05 vs. sham mice or sedentary mice (t-test). miR, microARN; premiR, precursor of microRNA; TAC, transaortic constriction; sed, sedentary; run, running mice.

We recently investigated how miR-199a controls the eNOS/NO pathway in the endothelium and highlighted that both mature strands of miR-199a were overexpressed in a mouse model of hypertension induced by chronic angiotensin II infusion (Joris et al., 2018). In this study, we first examined changes in miR-199a expression in vessels of mice developing pathological hypertrophy after transverse aortic constriction (TAC). We found a marked upregulation of both miR-199a-3p and miR-199a-5p in mesenteric arteries and aorta compared with sham-operated controls (**Figures 2A, B**; **Supplementary Figures 2A, B**). In striking contrast, these microRNAs were significantly downregulated in arteries from exercise-trained mice relative to sedentary counterparts (**Figures 2F, G** and **Supplementary Figures 2F, G**). Consistent with the established effects of exercise, the NOS/NO signaling pathway was upregulated in trained mice. Aortic tissues from exercised animals displayed increased phosphorylation of eNOS at Ser1177, concomitant with enhanced Akt phosphorylation at Thr308, relative to sedentary controls (**Supplementary Figures 3A, B**). In agreement with the increased influence of NO on vascular tone, eNOS inhibition produced a greater enhancement of phenylephrine-induced contractile responses in mesenteric arteries from exercised mice compared with sedentary animals (**Supplementary Figure 3C**). This corroborates our published data that repressed miR199a-3p and -5p associate with improved NO-dependent signaling and endothelial function.

To verify that the observed microRNA dysregulation was intrinsically vascular and not due to import from other tissues, we assessed the expression of its precursors and of a candidate transcriptional regulator. *Twist-1*, is a transcription factor that drives expression of a 7.9-kb non-coding RNA within the *Dynamin-3* intron, which contains the *miR-199a/miR-214* cluster (Lee et al., 2009). Consistently, *premiR-199a-2* and *Twist-1* mRNA levels were elevated in vascular samples from TAC mice compared with shams (**Figures 2D, E**; **Supplementary Figures 2D, E**), whereas both transcripts were reduced in the exercise group (**Figures 2I, J**; **Supplementary Figures 2I, J**). In contrast, *premiR-199a-1* expression remained unchanged (**Figures 2C, H**; **Supplementary Figures 2C, H**).

**Figure 3:**
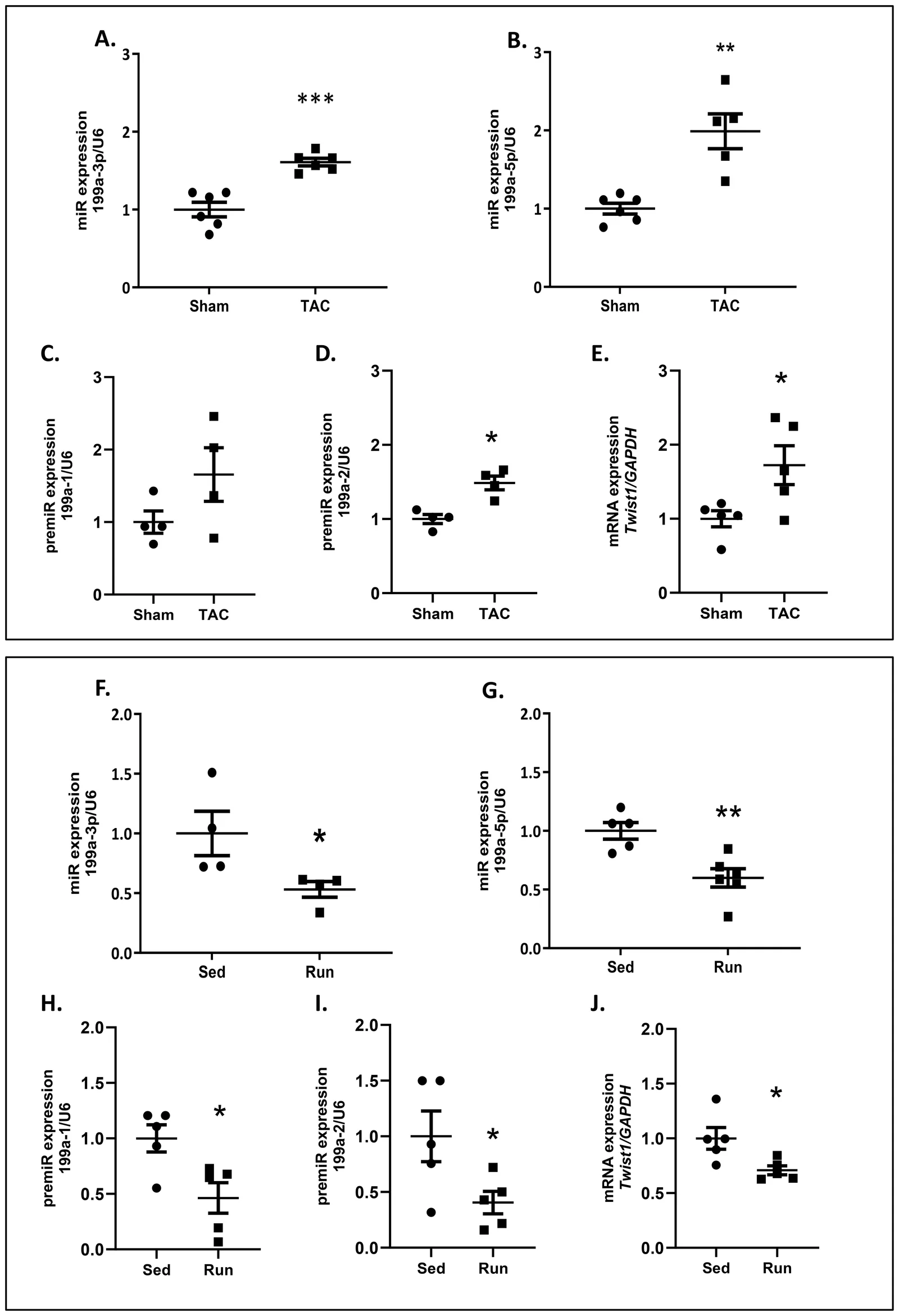
Hearts isolated from TAC mice (upper panel) and running mice (lower panel) exhibit opposite microRNAs 199a expression. MiR-199a-3p and miR-199a-5p expression were measured in heart samples from TAC **(A, B)** and running mice **(F, G)** using RT-qPCR. PremiRs expression and Twist1 expression were measured in heart samples from TAC **(C–E)** and running mice **(H–J)** using RT-qPCR. Results are expressed as mean ± SEM of 4–6 animals. *P. < 0.05, **P. < 0.01 and ***P. < 0,001 vs. sham mice or sedentary mice (t-test or Mann-Whitney when appropriated). miR, microARN; premiR, microRNA precursor; TAC, transaortic constriction; sed, sedentary; run, running mice.

We then investigated whether similar miR-199a alteration could be detected in the heart. In TAC mice, cardiac expression of miR-199a-3p and miR-199a-5p was markedly increased (**Figures 3A, B**). Conversely, both mature miRs strands were significantly downregulated in LV tissue of exercised mice compared with sedentary controls (**Figures 3F, G**).

Expression of *premiR-199a-1* and *premiR-199a-2* was increased in TAC hearts (**Figures 3C, D**) although this did not reach significance for *premiR-199a-1*. Consistent with the upregulation of premiR-199-2, *Twist-1* mRNA and protein expression were enhanced (**Figure 3E**; **Supplementary Figure 1G**). Interestingly, *Twist-1* mRNA and protein expression, as well as both *premiR-199a* transcripts were downregulated in cardiac tissue from exercised mice (**Figures 3H–J**; **Supplementary Figure 1H**), indicating differential regulation of this pathway in physiological *versus* pathological cardiac hypertrophy.

These findings indicate that miR-199a-3p/-5p and their regulatory components are oppositely modulated by pathological and physiological stimuli, suggesting a context-dependent control of vascular and cardiac remodeling.

### miR-199a-3p is a key regulator of cardiac and cardiomyocytes hypertrophy

**Figure 4:**
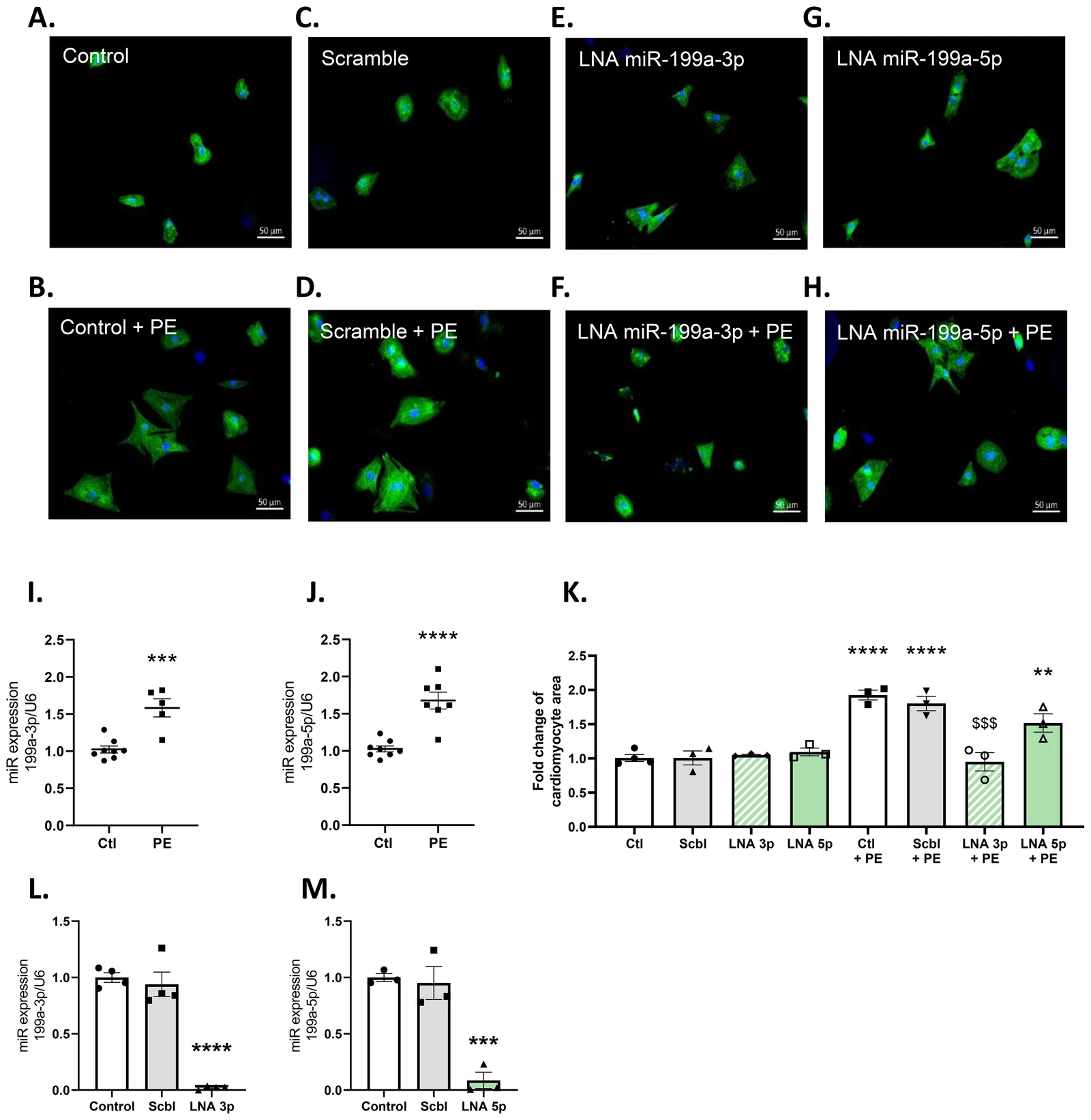
Downregulation of miR-199a-3p prevents the phenylephrine-induced hypertrophy in cardiomyocytes. Neonatal rat cardiomyocytes were transfected with scramble **(C, D)**, inhibitor of miR-199a-3p **(E, F)** or miR-199a-5p **(G, H)**. After 5 days, cardiomyocytes were treated with phenylephrine (PE, 50µM) for 24 hours **(B, D, F, H)**. The day after, miR modulation by PE **(I, J)** and inhibition of miR-199a-3p **(L)** and miR-199a-5p **(M)** were assessed by RT-qPCR. Surface area of cardiomyocytes was measured following α-actinin (green) and DAPI (blue) IF-staining **(K)**. Results are expressed as mean ± SEM of 3 independent experiments **P.<0,01, ***P.<0,001, ****P.<0.0001 vs. control cardiomyocytes **(A)**. $$$, P.<0.001 vs control + PE cardiomyocytes **(B)** (ANOVA).

**Figure 5:**
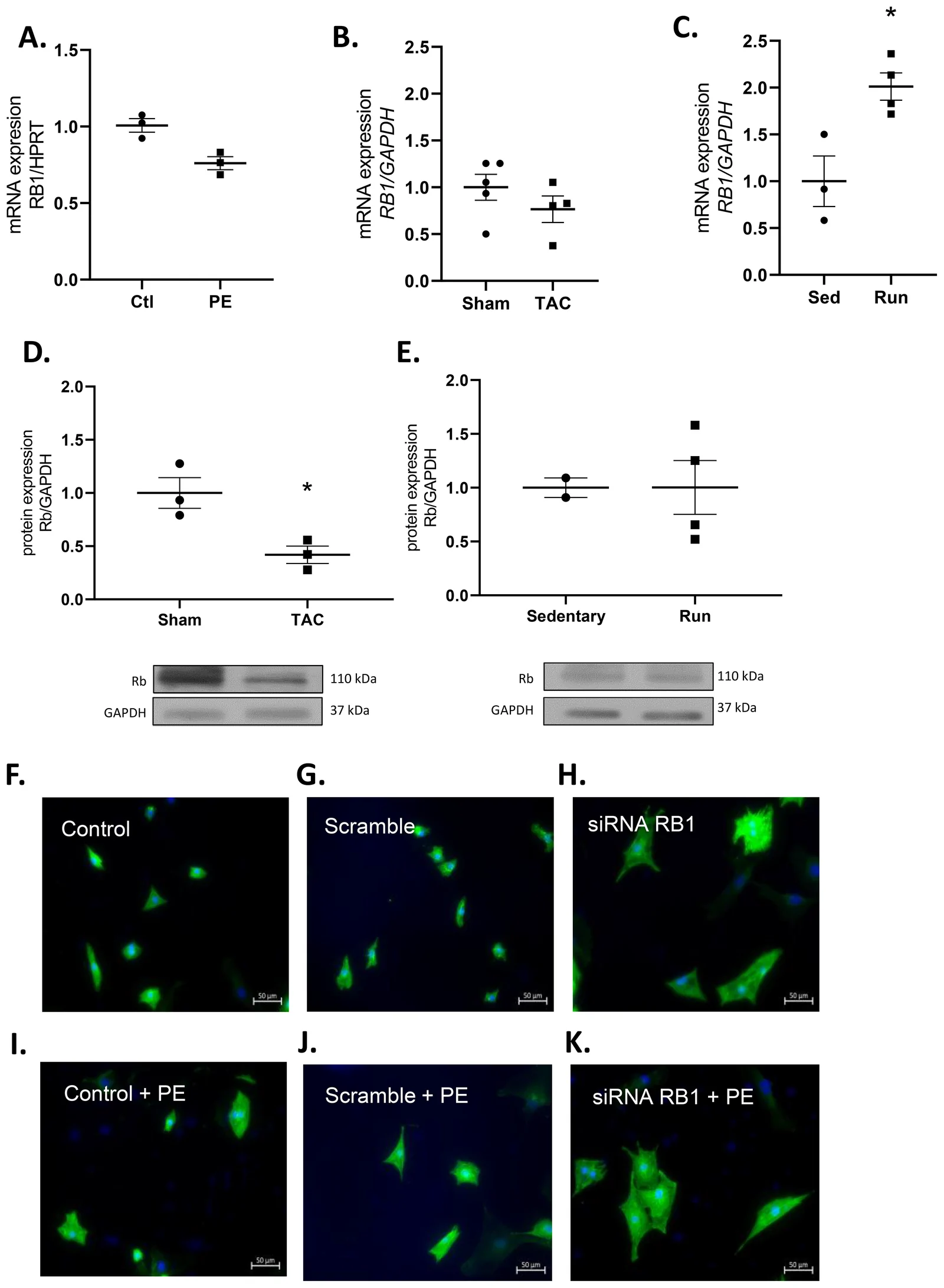
RB1 downregulation induces hypertrophy in cardiomyocytes. Neonatal rat cardiomyocytes were transfected with scramble **(G, J)** or siRNA targeting RB1 (50μM) **(H, K)**. After 5 days in culture, cardiomyocytes were treated with phenylephrin (PE, 50μM) for 24 hours **(I–K)**. The day after, mRNA expression of RB1 was assessed by RT-qPCR in cardiomyocytes treated with PE **(A)** and surface area of cardiomyocytes was highlighted following α-actinin (green) and DAPI (blue) IF-staining **(F–K)**. In addition to that, RB1 mRNA and protein expression were measured in heart samples of TAC **(B, D)** and running mice **(C, E)**. Results are expressed as mean ± SEM of 3 independent experiments. *, P.0,05, **, P.<0,01 vs. control (T-test or ANOVA when appropriated). RB1, retinoblastoma protein; PE, phenylephrine; TAC, transaortic constriction; sed, sedentary; run, running mice.

Given the opposite regulation of miR-199a strands in pathological *versus* physiological contexts, we next investigated whether these miRNAs directly modulate cardiomyocyte size. Rat neonatal cardiomyocytes were treated with phenylephrine (PE) for 24 h to model cardiac hypertrophy induced by elevated catecholamine levels. While PE promoted cardiomyocytes hypertrophy (**Figures 4A, B, K**), both miRs strands showed a significant up-regulation (**Figures 4I, J**), consistent with the pathological nature of the PE-induced response. Rat neonatal cardiomyocytes were then treated with LNA inhibitors against miR-199a-3p or -5p *versus* scrambled controls. Knockdown efficiency was verified (**Figures 4L, M**). As expected, PE increased cardiomyocyte surface area in scramble conditions as in controls (**Figures 4D, B**). Strikingly, inhibition of miR-199a-3p, but not miR-199a-5p, significantly prevented PE-induced hypertrophy in cultured cardiomyocytes (**Figures 4F, H**). Conversely, transfection with miR-199a-3p mimics induced hypertrophy in the absence of PE stimulation (**Supplementary Figure 4**), confirming its pro-hypertrophic nature. Given that these findings supported the role of miR-199a upregulation in promoting PE-induced pathological hypertrophy, we aimed to identify potential differences in the signaling events downstream of PE stimulation to discern pathways that could differentiate between TAC-induced and exercise-induced cardiac hypertrophy. We chose to focus on RB1 (Retinobastoma1) which is known as a target of miR-199a-3p (Lee et al., 2010), while an interaction with miR-199a-5p has also been reported, the evidence supporting this association is less consistent (Zhou et al., 2014). In addition to that, RB1 is also involved in the AMPK-mediated signal triggering E2F-dependent activity of cyclins and CDK1 associated with pathologic cardiac growth (Bischof et al., 2021). We found that RB1 expression is repressed in cardiomyocytes treated with PE (**Figure 5A**), while silencing of RB1 promotes rat neonatal cardiomyocyte hypertrophy (**Figures 5F–K**). Accordingly, both RB1 mRNA and Rb protein expression were lower in the heart of TAC mice in comparison to their controls (**Figures 5B, D**). Strikingly, the hypertrophied hearts of trained mice exhibited a two-fold increase in RB1 mRNA expression compared with those of sedentary controls (**Figure 5C**). In contrast, Rb protein expression remained unchanged after exercise (**Figure 5E**). Pointing to alternative pathways to drive exercise-induce cardiomyocytes growth.

### miR-199a-5p targets metabolic regulators in cardiac tissue

**Figure 6:**
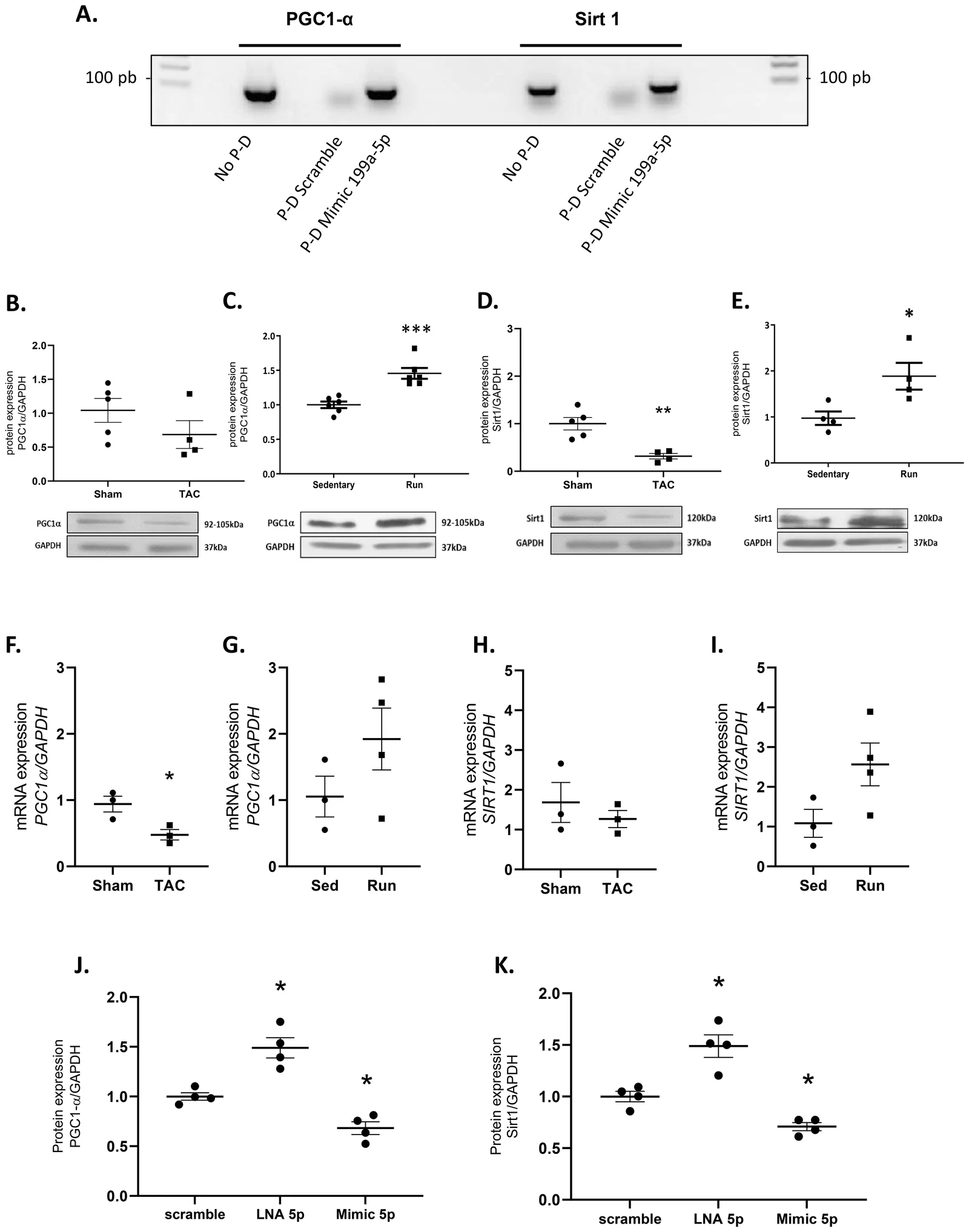
Sirt1 and PGC1α are direct targets of miR-199a-5p in heart. Pull-Down (P-D) was realized on cardiomyocytes transfected with biotinylated scramble or mimic of miR-199a-5p **(A)**. Protein expression levels of PGC1α and Sirt1 were detected by Western Blotting in heart of TAC **(B, D)** and running **(C, E)** mice; and mRNA of PGC1α **(F, G)** and Sirt1 **(H, I)** were measured by RT-qPCR in heart of both running and TAC mice. Protein expression of PGC1α and Sirt1 was also measured in isolated cardiomyocytes transfected with mimics or inhibitor of miR-199a-5p **(J, K)** and quantified. Results are expressed as mean ± SEM of 4–6 animals or 4 individual experiments on cells. *P. < 0.05, ** and ***P. < 0,001 vs. sedentary mice or scramble (t-test or ANOVA when appropriated). Run, running mice; TAC, trans-aortic constriction.

In our quest to understand the mechanisms driving physiological cardiac hypertrophy, we turned our attention towards other validated targets of miR-199a, namely *Sirt-1* and *PGC-1α* (Rane et al., 2009; Jia et al., 2013; Lin et al., 2017; Chen et al., 2018). This direct interaction was confirmed by pull-down assays in cultured neonatal cardiomyocytes (**Figure 6A**). *In vivo*, chronic exercise induced a significant upregulation of both *PGC-1α* and *Sirt-1* protein expression (**Figures 6C, E**). In contrast, those proteins were decreased in TAC mice (**Figures 6B, D**). A slight increase in *PGC-1α* and *Sirt-1* mRNAs was also observed in the heart of running mice, while those mRNAs were downregulated in TAC mice (**Figures 6F–I**). More interestingly, inhibition of miR-199a-5p in cardiomyocyte cultures increased *PGC-1α* and *Sirt-1* expression, whereas overexpression using miR-199a-5p mimics produced the opposite effect (**Figures 6J, K**), supporting a direct regulatory link consistent with *in vivo* findings. Of note, SIRT1 upregulation can reinforce PGC1α activity since SIRT1 is known to deacetylate and activate PGC-1α.

**Figure 7:**
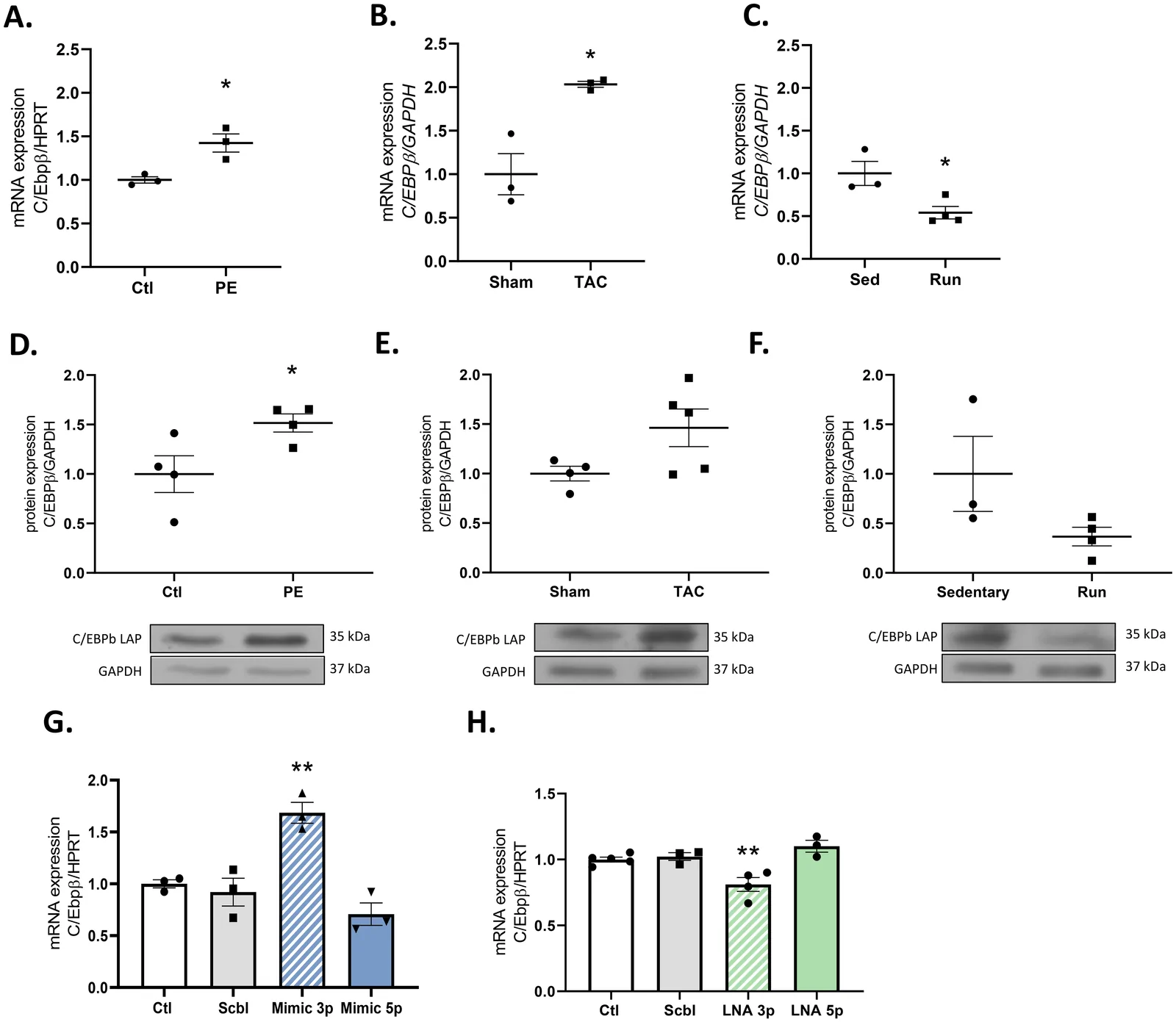
C/EBPβ is modulated by miR-199a-3p, and a key regulator of cardiac hypertrophy. Neonatal rat cardiomyocytes were transfected with scramble, mimic or inhibitor of miR-199a-3p or -5p (50nM) **(G, H)**. After 5 days in culture, cardiomyocytes were treated with phenylephrin (PE, 50μM) for 24 hours. The day after, mRNA expression of C/EBPβ was assessed by RT-qPCR in cardiomyocytes treated with PE **(A)**, transfected with mimic **(G)** or LNA **(H)**. C/EBPβ LAP protein expression was also assessed following PE treatment **(D)**. In parallel, C/EBPβ mRNA and protein expression were measured in heart samples of TAC **(B, E)** and running mice **(C, F)**. Results are expressed as mean ± SEM of 3 independent experiments *, P.0,05, **, P.<0,01 vs. control (T-test or ANOVA when appropriated). C/EBPβ, CCAAT/enhancer-binding protein beta; PE, phenylephrine; TAC, transaortic constriction; sed, sedentary; run, running mice.

Interestingly, PGC1α induction was previously reported to be coupled with a reduction in CCAAT/enhancer binding protein (C/EBP) β expression which is documented to support physiological cardiac hypertrophy and provide resistance to cardiac failure upon pressure overload (Boström et al., 2010). Consistently, *C/EBPβ* mRNA and protein expression were downregulated in hearts from exercised mice (**Figures 7C, F**) and upregulated in TAC (**Figures 7B, E**) hearts in comparison to their respective controls. Moreover, in cardiomyocyte cultures, PE treatment also induced *C/EBPβ* expression (**Figures 7A, D**). Our data indicate that the regulation of C/EBPβ by miR-199a-3p is indirect and involves the mTOR signaling pathway. While transfection with miR-199a-3p mimics, but not miR-199a-5p mimics, increased C/EBPβ expression, whereas inhibition of the 3p strand using LNA oligonucleotides reduced C/EBPβ levels (**Figures 7G, H**). Conversely, miR-199a-3p overexpression decreased mTOR expression, while miR-199a-3p inhibition increased mTOR levels (**Supplementary Figures 5A, B**). Importantly, inhibition of mTOR with specific siRNA reduced C/EBPβ LIP expression (**Supplementary Figures 5C, D**). Taken together, these findings suggest that miR-199a-3p regulates C/EBPβ indirectly through modulation of mTOR signaling.

**Figure 8:**
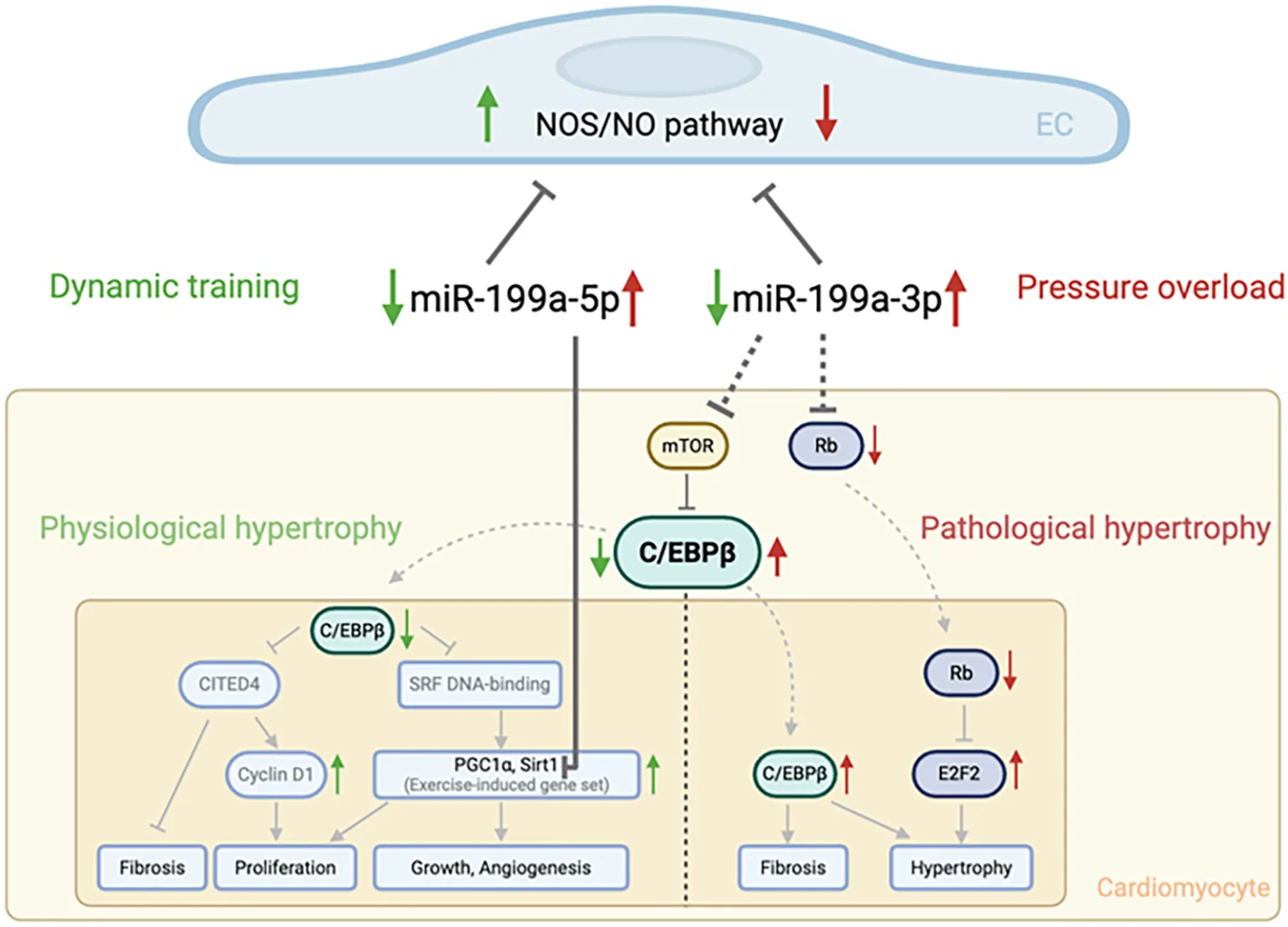
Proposed model of the coordinated regulation of cardiac adaptation by miR-199a. Left (green): Dynamic exercise induces coordinated repression of miR-199a-3p and miR-199a-5p, promoting physiological cardiac and endothelial adaptations. Repression of miR-199a-3p is associated with reduced C/EBPβ expression, favoring metabolic adaptations underlying physiological remodeling, whereas repression of miR-199a-5p relieves inhibition of Sirt1 and PGC-1α, supporting mitochondrial metabolism, angiogenesis, endothelial function, and adaptive cardiomyocyte growth. Right (red): Pressure overload elicits opposite regulation of both mature miR-199a strands, resulting in increased miR-199a-3p and miR-199a-5p expression and reciprocal modulation of C/EBPβ, Sirt1, and PGC-1α, thereby promoting pathological cardiac hypertrophy, metabolic dysfunction, fibrosis, and adverse cardiac remodeling. Together, these findings suggest that both mature arms of miR-199a act in concert to coordinate the morphological and metabolic adaptations underlying physiological and pathological cardiac hypertrophy.

Altogether our results point to a major role of miR-199a-3p in the control of *C/EBPβ* expression that drives metabolic and morphologic adaptations of cardiac tissue in response to pressure or volume overload while the opposite mature strand, miR-199a-5p directly regulates PGC1α and Sirt-1 to promote mitochondrial biogenesis and oxidative metabolism in the context of exercise (see **Figure 8**).

## Discussion

Our findings establish miR-199a as a key molecular link between physiological and pathological cardiac remodeling and reveal a coordinated regulatory network involving both miR-199a strands and multiple downstream effectors. Mature forms of miR-199a (miR-199a-3p and miR-199a-5p) are key regulators of cardiovascular homeostasis (Catalucci et al., 2008; Neppl and Wang, 2014). In this study, we compared murine models of dynamic training and of pressure overload by aortic banding in order to evaluate whether changes in miR-199a abundance could account for cardiac and vascular adaptations in these settings. Mostly from *in vitro* data, our previous work has revealed that both mature miR-199a strands acts redundantly to regulate the eNOS/NO pathway and endothelial function (Joris et al., 2018). Here, we extend these observations *in vivo* in more complex and relevant models reflecting physiological and pathological conditions. Although miR-199a-3p and miR-199a-5p have distinct targets and biological functions, their expression is primarily driven by shared transcriptional regulation of the miR-199a precursor. Under pathological mechanical stress (TAC), transcription of the miR-199a locus is globally increased, leading to parallel elevation of both mature strands. Conversely, endurance training suppresses precursor transcription, resulting in coordinated downregulation of both miR-199a-3p and -5p. More importantly, we demonstrated that both mature miR strands participate in the morphologic and metabolic adaptations encountered in physiological and pathological cardiac remodeling, positioning miR-199a at the crossroad between CV health and diseases.

The heart adapts to increased workload through hypertrophic growth, irrespective of whether the initial stimuli is physiological or pathological. While this hypertrophic response appears similar, the underlying processes differ markedly particularly regarding fibrosis development, fetal gene expression reprogramming or the presence of angiogenic support. For physiological cardiac hypertrophy, if the picture is clear in human (Fagard, 2003), literature is a bit less consistent as regards to exercise-induced hypertrophic growth effects in rodents. Types, duration and intensity of exercise training (Wang et al., 2010), species and genetic background (Lerman et al., 2002) could pertain for these discordant observations. Among the constant features, the absence of a shift from α- (the adult form) to β-MHC isoform (the fetal form) and the absence of modulation of SERCA2a expression, confirmed that the contractile properties of the hearts of trained mice are not negatively altered as observed in the heart of the pressure overload model.

To our knowledge, this is the first time that a down-regulation of miR-199a-3p and -5p is highlighted in cardiac (and vascular) tissue of mice submitted to a protocol of dynamic training and exhibiting physiological hypertrophy. This observation is in dramatic contrast with what is observed in pathological cardiac hypertrophy. Surprisingly, only miR-199a-5p was modulated in the plasma in our two mice models (data not shown), suggesting that the regulation of the two arms observed either in cardiomyocytes or vessels is intrinsic to these cell/tissue types. Other studies have already pointed out miR-199a-5p in cardiac tissue during pathological hypertrophy (Rane et al., 2010; Song et al., 2010), but here we show that miR-199a-3p is also concerned to more specifically promote cardiomyocyte growth and probably fibrosis. Our work provides evidence for a differential contribution of the two arms of pre-miR-199a to the regulation of cardiomyocyte hypertrophy. Transfection of neonatal rat cardiomyocytes with an inhibitor specifically targeting miR-199a-3p prevented phenylephrine (PE)-induced hypertrophy, whereas overexpression of miR-199a-3p through a mimic was sufficient to induce a marked increase in cell size. These findings support a pro-hypertrophic role for miR-199a-3p. They are also consistent with previous functional screening studies identifying miR-199a as one of the most potent microRNAs capable of inducing cardiomyocyte proliferation and cardiac regeneration after myocardial injury, further supporting its central role in regulating cardiomyocyte growth and plasticity (Eulalio et al., 2012; Lesizza et al., 2017). In contrast, miR-199a-5p appears to exert little to no effect under these experimental conditions, suggesting functional divergence between the two isoforms.

Our results also identify opposite regulation of C/EBPβ in physiological *versus* pathological hypertrophy. We further highlight an interaction between miR-199a-3p and C/EBPβ, which occurs indirectly via the mTOR pathway (see **Supplementary Figure 5**), a known target of this microRNA. Again, no substantial involvement of miR-199a-5p was detected in this regulatory axis, reinforcing the notion of functional specialization of the 3p arm. Downregulation of C/EBPβ has been consistently observed in the context of exercise, independent of the modality of training, whether dynamic training or resistance/strength exercise. Under resting conditions, C/EBPβ represses *CITED4* expression. During exercise downregulation of C/EBPβ alleviates this repression, enabling *CITED4* upregulation, which in turn promotes cardiomyocyte growth, proliferation (Boström et al., 2010), and repression of fibrosis (Bezzerides et al., 2016).

In parallel, we highlighted the role for RB1 in pathological hypertrophy. Both PE-treated cardiomyocytes and hearts subjected to transverse aortic constriction (TAC) show reduced RB1 mRNA expression. Moreover, RB1 inhibition via siRNA in cardiomyocytes was sufficient to induce hypertrophy, supporting a protective function of RB1. Although RB1 has been reported as a potential target of both miR-199a-3p and miR-199a-5p (Lee et al., 2010; Zhou et al., 2014), LNA-mediated inhibition of these microRNAs did not significantly alter RB1 mRNA levels in our hands (data not shown), suggesting that the observed post-transcriptional regulation may depend on context-specific or more subtle mechanisms, not recapitulated in the PE-treated cardiomyocytes.

Beyond transcriptional regulation, Rb protein activity is also modulated post-translationally through phosphorylation by Cyclin D–cdk4/6 and Cyclin E–cdk2 complexes, leading to its dissociation from E2F and promoting cell-cycle entry (Weinberg, 1995; Lundberg and Weinberg, 1998; Pasumarthi et al., 2005; Strauss et al., 2012). Previous studies have shown that Cyclin D expression increases during both physiological (Boström et al., 2010) and pathological hypertrophy (Li et al., 1998; Busk, 2002; Tamamori-Adachi et al., 2002). However, our data suggest a more controlled regulation of the RB1/Rb axis during exercise-induced hypertrophy, in contrast to the dysregulation observed in pathological settings.

The miR-199a/214 cluster has been shown to repress cardiac PPARδ expression, thereby contributing to a metabolic switch from predominant fatty acid utilization in the healthy myocardium toward increased glucose dependence at the onset of heart failure (el Azzouzi et al., 2013). In our model of dynamic training, we observe a downregulation of miR-199a in the heart accompanied by an upregulation of PGC-1α, These findings are consistent with the work of Li et al., who used a cardiomyocyte-specific miR-sponge to repress miR-199a and demonstrated that a modest reduction (20–30%) in miR-199a levels promotes physiological cardiac hypertrophy associated with increased PGC-1α expression. Our data further highlighted the specific interactions between miR-199a-5p and PGC-1α and SIRT-1. The elevation of PGC-1α is particularly noteworthy, as this coactivator is a key regulator of cardiomyocyte metabolism and a major driver of mitochondrial biogenesis (Caravia et al., 2018). In line with the concept of differential regulation of miR-199a in pathological versus physiological hypertrophy, exercise induces PGC-1α activation in the heart, whereas pathological stimuli suppress its activity (Barger et al., 2000). Together, these observations further support the notion that controlled reduction of miR-199a is associated with adaptive, metabolically favorable remodeling in response to exercise, in contrast to its maladaptive regulation in disease.

Although our data revealed striking differences in collagen deposition between TAC and control hearts and showed that exercise training prevents normal age-related cardiac fibrosis, we did not investigate whether miR-199a contributes to these distinct fibrotic responses, this is a limitation of the present study. Previous studies have already reported a profibrotic role for miR-199a-3p in pathological settings (Liang et al., 2018; Zalivina et al., 2023). Interestingly, miR-199a-3p has not been associated with pregnancy-induced physiological cardiac hypertrophy (Szczerba et al., 2020), further supporting the notion that its functions may be context-dependent and vary according to the nature of the cardiac stimulus. Future studies specifically addressing cardiac fibrosis across distinct adaptive and maladaptive remodeling paradigms will be required to clarify this aspect.

Our previous work identified miR-199a-3p and -5p as central components of a redundant regulatory network repressing eNOS activity and NO bioavailability in the endothelium (Joris et al., 2018). Consistently, blocking either strand, individually or together, enhanced NO production, NO-dependent relaxation, and circulating NO levels *in vivo*. Here, we show that chronic dynamic wheel training promotes the eNOS/NO pathway while inducing a downregulation of both miR-199a strands, in opposition pressure overload promoted miR-199a up-regulation and potentially endothelial dysfunction. Interestingly, the dysregulation of the miR-precursors observed in both vascular and cardiac tissues suggests the presence of tissue-specific regulatory mechanisms in each compartment. Given the close functional interplay between the vasculature and the myocardium, we may infer that miR-199-driven endothelial dysfunction would worthen cardiac condition in the context of pressure overload while miR-199 associated promotion of the NO pathway would reinforce a physiological cardiac phenotype.

Taken together, our findings indicate that cardiac and endothelial adaptations to dynamic exercise or pressure overload are closely linked to coordinated and opposite modulations of both strands of miR-199a that drive endothelial function, cardiomyocyte growth and expression of C/EBPβ, the main architect of metabolic adaptations in response to volume or pressure overload. In addition, our work extends the notion that both strands of a same miR might act redundantly to promote cellular and tissular adaptations in response to external stimuli (**Figure 8**).

## Data Availability

The raw data supporting the conclusions of this article will be made available by the authors, without undue reservation.
